# Preduodenal Portal Vein Associated with Duodenal Obstruction of other Etiology: A Case Series

**DOI:** 10.21699/jns.v5i4.341

**Published:** 2016-10-10

**Authors:** Punit Srivastava, Mishraz Shaikh, Bilal Mirza, Richa Jaiman, Muhammad Arshad

**Affiliations:** 1 Department of Pediatric Surgery, S N Medical College Agra; 2 Department of Pediatric Surgery, Liaquat National Hospital, Karachi, Pakistan; 3 Department of Pediatric Surgery, Children Hospital Faisalabad

**Keywords:** Preduodenal portal vein, Duodenal Atresia, Polysplenia, Situs inversus, Heterotaxy

## Abstract

DuodenalPreduodenal portal vein is a rare and interesting entity which often causes duodenal obstruction. It is also associated with other congenital anomalies. We report here three cases of preduodenal portal vein associated with other anomalies causing duodenal obstruction not related to direct compression by portal vein itself.

## INTRODUCTION

Preduodenal portal vein (PDPV) is a rare anomaly first described by Knight in 1921. [1]It may be an incidental finding while operating for duodenal obstruction. It is associated with other anomalies especially heterotaxy and polysplenia syndrome. [2,3] It may cause duodenal obstruction by directly compressing the lumen of the duodenum [2] or the associated anomalies may cause duodenal obstruction. [3] Herein, we report three neonates with PDPV presented with duodenal obstruction not related to PDPV itself.

## CASE SERIES

**Case 1:**

A 3 day-old male baby referred with a history of non-bilious vomiting since first day of life. The neonate was born full term by normal delivery at periphery hospital. There was no abdomen distention or signs of sepsis. Plain abdomen radiograph showed a double bubble sign, with distal gasless abdomen. Clinical diagnosis of duodenal atresia was made. At laparotomy, malrotation with midgut volvulus was found. The bowel was viable and Ladd’s procedure was performed. Further exploration revealed portal vein crossing first part of duodenum anteriorly (Fig.1). Nasogastric tube was advanced which passed easily beyond PDPV area but an obstruction was found at second part of duodenum. A lateral duodenotomy was performed which identified a thick duodenal web obstructing the lumen. The Ampulla of Vater was identified distal to web. Duodenal web excised circumferentially and mucosa was over sewn continuously with Vicryl 5/0 suture for homeostasis; duodenum was closed transversely in single layer interrupted suture with PDS 4/0. Postoperative recovery was uneventful. At 2-month follow-up the child is doing well and gaining weight. 

**Figure:**
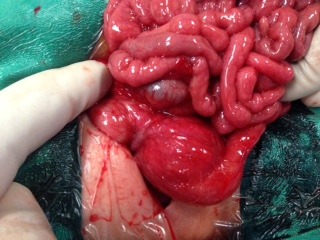
Figure 1: Case 1- showing preduodenal portal vein crossing anterior to the dilated duodenum. The duodenum beyond level of PDPV is also dilated indicating non-obstructing type of PDPV.

**Case 2:**

An 18-day-old full term male baby, with a birth weight of 2.5 kg, presented with recurrent bilious vomiting since birth. On auscultation of chest, heart was found on right side. Plain abdomen radiograph showed gastric bubble on the right side of abdomen with paucity of gas in the rest of abdomen. Ultrasonography showed the liver and the gallbladder on the left side of abdomen. At surgery, the stomach and spleen were found on the right side, while the liver was on the left side. The duodenal loop was on the left side. There was a pre-duodenal portal vein crossing over proximal duodenum which was non-obstructing (Fig.2). It was confirmed by pushing normal saline through nasogastric tube. The cause of bilious vomiting was malrotation. Ladd’s procedure was performed in reverse fashion and bowel patency was checked. The postoperative recovery was uneventful. Patient is well on follow-up.

**Figure:**
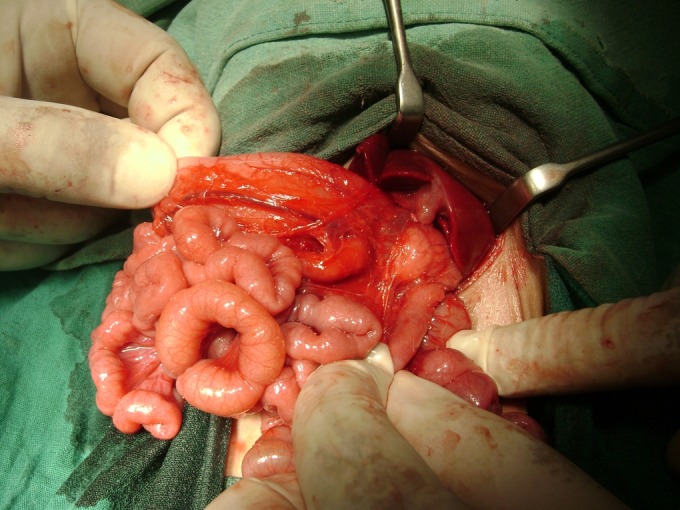
Figure 2: Case 2- PDPV crossing anteriorly to duodenum. Heterotaxy can be appreciated (stomach right sides and gallbladder left sided).

**Case 3:**

A 3-day old male neonate presented with bilious vomiting and failure to pass meconium since birth. Examination revealed a sick looking dehydrated neonate with mild upper abdominal distension. The neonate was initially resuscitated for dehydration and a nasogastric tube was passed which revealed bilious aspirate. An x-ray abdomen was performed which revealed right sided dilated gastric bubble and another shadow in the mid abdomen (reverse double bubble). The laboratory parameters revealed low platelets (20x103/cc). CRP was mildly raised. Blood culture did not yield any culture. The child required platelet transfusion and broad spectrum antibiotics. On improvement in laboratory and clinical parameters, an exploratory laparotomy was performed which showed heterotaxy of abdominal viscera (right sided stomach, intraperitoneal dilated duodenum with portal vein running over the duodenum, left sided gallbladder, right sided polysplenia [1 full spleen and 6-7 small splenules], and retroperitoneal transverse colon) (Fig.3,4). A bypass duodeno-duodenostomy was planned and on opening the proximal duodenum, a nasogastric tube was passed to check distal obstruction which revealed duodenal atresia about 1.5cm distal to the level of PDPV crossing the duodenum. The distal incision was made just distal to the atresia and diamond shaped duodeno-duodenostomy was fashioned. The postoperative course remained stormy. The patient again went into septicemia and succumbed on 5th postoperative day.

**Figure:**
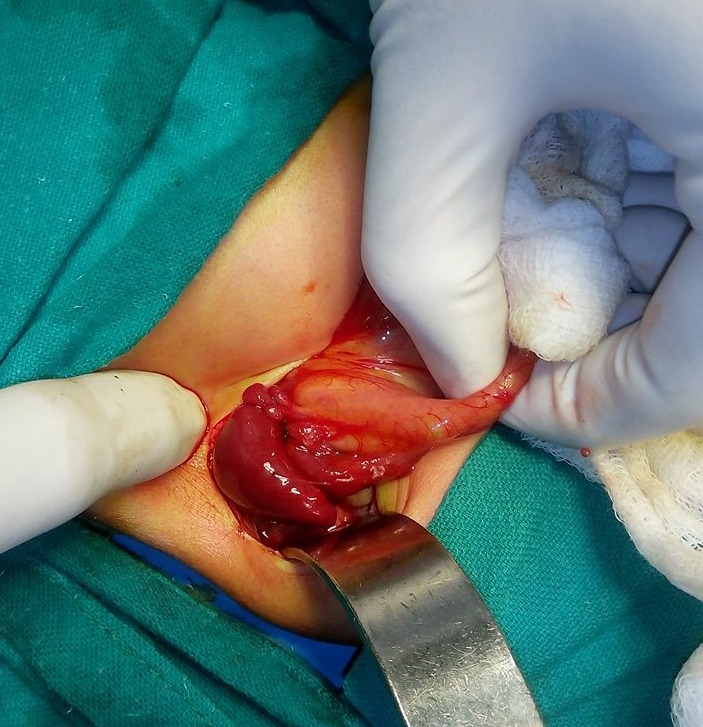
Figure 3: Case 3- Right sided stomach and polysplenia.

**Figure:**
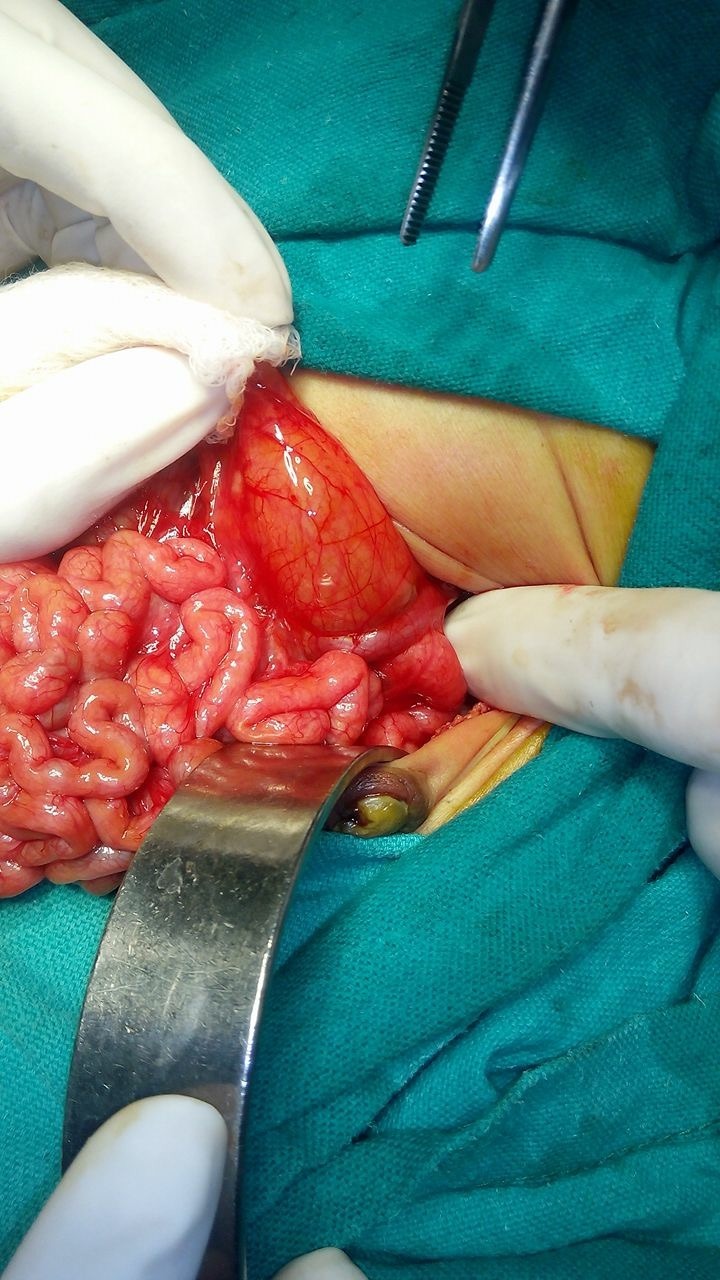
Figure 4: Case 3- PDPV crossing duodenum anteriorly.

## DISCUSSION

Preduodenal portal vein is a rare entity with duodenal obstruction being it’s most common clinical presentation although in less than half of these cases PDPV was directly responsible for duodenal obstruction. Majority of the reported cases have associated anomalies responsible for duodenal obstruction. These anomalies are duodenal atresia or web, annular pancreas or anomalies of rotation and fixation. [3, 4] In our series, PDPV did not cause duodenal obstruction. In our first case the cause of duodenal obstruction was associated duodenal web although malrotation and midgut volvulus were also present. In our second case, the duodenal obstruction was caused by malrotation. In our third case, the duodenal obstruction was by duodenal atresia.

PDPV usually associated with other anomalies such as; duodenal, cardiac, biliary, pancreatic and splenic anomalies [2, 5]. Yi et al [6] reviewed the largest series of PDPV cases and found 323 reported cases of PDPV with multiple associated anomalies including intestinal malrotation in 64%, situs inversus in 26%, duodenal and pancreatic anomalies in 26%and 22%, respectively. In our series, our first case was associated with duodenal web and malrotation with volvulus which is itself a very rare combination. In our second case PDPV was associated with malrotation and situs inversus totalis; in our third case it was associated with situs inversus abdominus, duodenal atresia, reverse rotation of intestine, and polysplenia syndrome.

Preduodenal portal vein, when unrecognized, is at risk to injury during surgery in the vicinity such as during cholecystectomy, gastrectomy, porto-enterostomy, and pancreatectomy.[7] A vigilant surgeon can prevent these complications by recognizing this anomaly early. The second issue during surgery in a patient with PDPV is identifying exact etiology of duodenal obstruction.[3,8,9] Peroperative negotiation of nasogastric tube across the level of PDPV can identify the nature of obstruction. In all of our cases, the nasogastric tube passed beyond the level of PDPV and duodenal obstruction was caused by other congenital associated anomalies.

In conclusion, heterotaxy with duodenal obstruction should raise the clinical suspicion of PDPV. On the contrary, presence of PDPV at surgery should alert the surgeon to look for other associated anomalies that can lead to duodenal obstruction. A nasogastric tube is always helpful in ruling out obstruction related to PDPV.

## Footnotes

**Note:** All the first three authors share equal first authorship.

**Source of Support:** Nil

**Conflict of Interest:** One of author is editor of the journal but the manuscript is handled on merit by the guest editor of special issue.
